# Localized Ewing sarcoma of the tibia

**DOI:** 10.1186/2045-3329-3-2

**Published:** 2013-02-04

**Authors:** Takeshi G Kashima, Nimali G Gamage, Uta Dirksen, Christopher LMH Gibbons, Simon J Ostlere, Nicholas A Athanasou

**Affiliations:** 1 Nuffield Department of Orthopaedics, Rheumatology and Musculoskeletal Sciences, University of Oxford, Nuffield Orthopaedic Centre, Oxford OX7HE, UK; 2 Department of Pediatric Hematology and Oncology, University Children’s Hospital Muenster, Muenster, Germany

**Keywords:** Ewing sarcoma, Bone tumour, Malignant, Prognosis

## Abstract

Ewing sarcoma (ES) is a high-grade malignant primary round cell tumour of bone in which there is commonly extension into extraosseous soft tissues at the time of diagnosis. This report details the clinical, radiological and pathological features of a case of ES of the tibia in which there was extensive osseous involvement but no infiltration beyond the periosteum into surrounding soft tissue. We also record the findings of one other ES case that exhibited similar behaviour. Both cases were male, involved the tibia and had the characteristic t (11;22) (q24;q12) translocation. No recurrence of tumour or metastasis has been seen in these two cases, both of which have had 6 years follow-up. Our findings indicate that there is heterogeneity in the behaviour of ES and show that localized ES is associated with a good prognosis.

## Introduction

Ewing sarcoma (ES) is a malignant tumour of bone which is composed of small round tumour cells [1,2]. It accounts for 6-8% of all primary malignant bone tumours and predominantly affects children, adolescents and young adults. It occurs rarely in patients older than 30 years and is more common in males than females (1.3:1). ES may arise in any bone. In long bones, the femur and tibia are most commonly affected [2]. Patients usually present with pain and swelling but pathological fracture may occur and some patients exhibit osteomyelitis-like systemic features. Molecular genetic studies have shown that in over 90% of cases ES tumour cells exhibit a characteristic reciprocal chromosomal translocation ie: t(11;22)(q24;q12) which results in fusion of the *EWS* gene and the *FLI*-1 gene [3].

ES is highly aggressive tumour which grows rapidly, causing extensive destruction of cancellous and cortical bone. In long bones, the tumour commonly involves the diaphysis and metaphysis with the epiphysis affected in only 2% of cases; radiologically, there is extensive permeative or moth eaten bone destruction and a soft tissue mass is seen in approximately 90% of cases at the time of diagnosis [4]. This report describes in detail a case of ES in which there was extensive ES involvement of the shaft and metaphysis of the tibial bone with erosion of the bone cortex, but unusually no involvement of soft tissues beyond the periosteum. We also describe the findings in one other case of ES of the tibia arising in a male that behaved similarly.

### Case report

#### Case 1

A 36-year old male presented with a 5 year history of left sided shin pain to his general practitioner. A plain radiograph taken at the time showed no bone or soft tissue abnormality. He presented again a few times with recurrent left leg and forefoot discomfort, particularly on exercise, before, 4 years later, complaining of more persistent severe shin pain, including at night. On clinical examination there was no bone or soft tissue swelling of the left leg. There was no other significant medical history and the patient was otherwise well. Haematological and biochemical investigations were normal, including white cell count, ESR and CRP.

**Figure 1:**
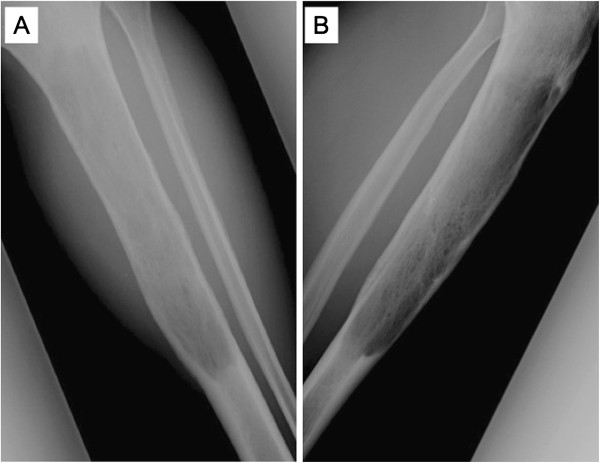
Case 1: A) Frontal and B) lateral radiographs of the tibia showing an expansile permeative lytic lesion involving the proximal tibial diaphysis.

**Figure 2:**
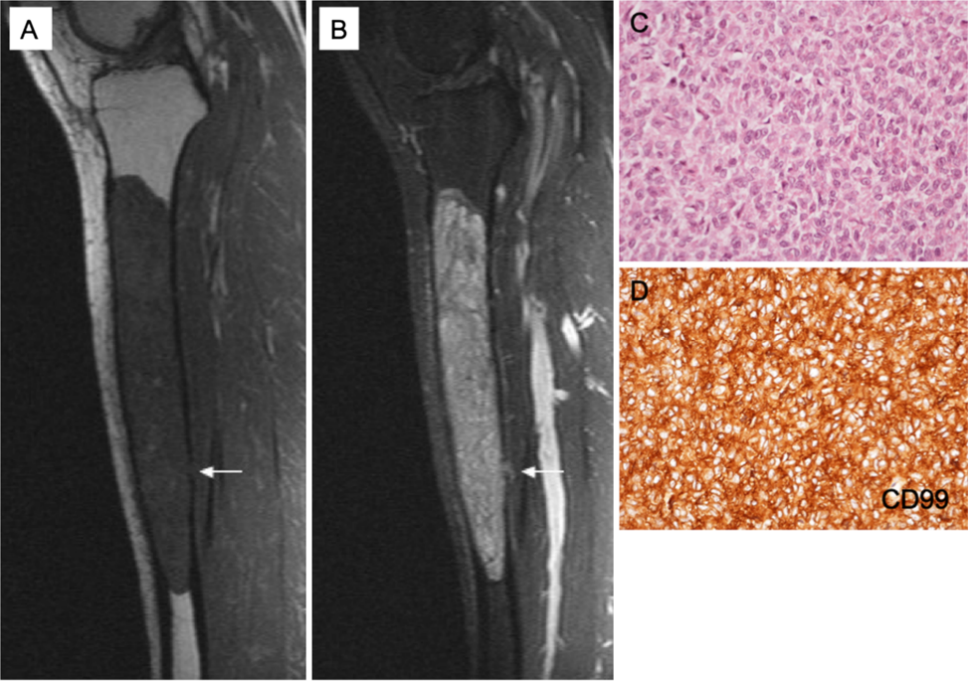
**Case 1: (A) T1-weighted and B) STIR sagittal MRI images showing a mildly heterogeneous, well-defined, expansile intramedullary lesion that is confined within the bone with the exception of a nubbin of tumour that has breached the posterior cortex (arrowed). **(**C**) Biopsy histology shows a malignant round cell tumour. (**D**) Tumour cells strongly express CD99.

Plain radiographs taken at this time showed a large expansile permeative lytic lesion involving the proximal half of tumour of the left tibial diaphysis (Figure 1). MRI demonstrated an intramedullary lesion showing predominantly high signal on the STIR sequence and low signal on the T1- weighted sequence (Figure 2). The lesion had a mildly heterogeneous appearance with scattered areas of ill-defined high signal on the T1 -weighted images. The proximal and distal margins of the lesion were well defined. A small nubbin of tumour measuring 0.5 cm in diameter was seen to extend into the posterior cortex of the distal third of the lesion. The lesion was otherwise contained within the bone.

Histopathology of a biopsy of the lesion showed a solid proliferation of tumour cells with plump cytoplasm and round vesicular or hyperchromatic nuclei (Figure 2C). Scattered cells had a vacuolated cytoplasm containing glycogen. Occasional typical mitotic activity was noted. The lesion was well-vascularized. The tumour appeared to infiltrate between bone trabeculae. Immunohistochemistry showed strong staining of the tumour cells for vimentin and CD99 (Figure 2D). The tumour cells did not express cytokeratin, EMA, HMB45, S100, CD45, CD20, CD31, CD34, Factor 8, podoplanin, muscle/smooth muscle actin, desmin or NB84a. There was a high proliferating fraction was noted on KI-67 staining.

Radiological and histological features indicated that this was an aggressive small round cell tumour that appeared to be confined to bone. The presence of glycogen-containing CD99+ cells pointed to a diagnosis of ES [2]. Molecular genetic investigations to confirm an EWS rearrangement were attempted on the biopsy material but were unsuccessful. Although the results pointed to a ES-like round cell tumour of bone, the absence of a soft tissue mass in the face of extensive intraosseous involvement was thought to be unusual for this entity. The patient was also noted to be somewhat outside the typical age range for ES. Staging studies including MR of the left lower limb, bone scan and chest CT showed no evidence of metastasis. After discussion with the patient, a segmental resection of the tibia to include the lesion and a margin of uninvolved bone was carried out. Reconstruction of the defect was carried out with a right vascularised free fibular graft and plating with stabilization of the left tibia with a 16 hole AO condylar plate and screw fixation.

**Figure 3:**
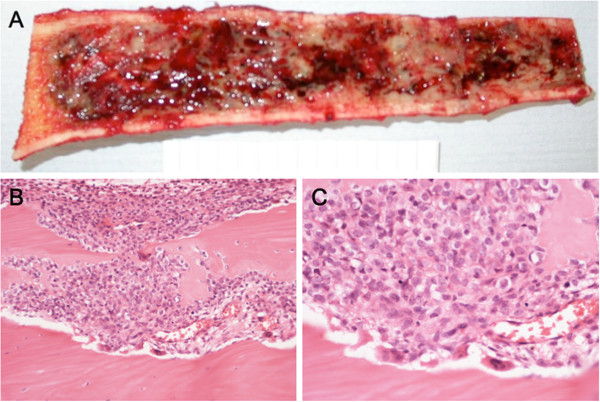
**Case 1: (A) The resected segment of the tibia shows extensive medullary involvement by ES. **(**B**), (**C**) Histology of the resection showing ES spread through medullary bone and erosion of the bone cortex.

The resected segment of tibia was grossly expanded and had a thickened cortex. Cut surface revealed a large haemorrhagic tumour 18x4x4cm which filled the medulla of the tibial shaft and metaphysis (Figure 3A); the tumour did not appear to extend through the bone cortex. Histologically, there was a solid proliferation of tumour cells with small round nuclei containing fine chromatin and scanty clear or eosinophilic cytoplasm. Tumour cells contained glycogen and there was little reticulin formation within the tumour. The tumour was well- vascularized and there were focal areas of haemorrhage within the tumour. There was infiltration of cancellous bone, much of which was undergoing osteoclastic resorption (Figure 3B). The tumour extended focally into the bone cortex reaching the outer surface but not penetrating through the periosteum into surrounding soft tissue (Figure 3C). There was no evidence of matrix formation by tumour cells. Cytogenetic investigation using interphase FISH revealed the presence of an EWSR1 rearrangement in keeping with a t(11;22)(q24;q12) translocation.

Taken together, the morphological, immunophenotypic and molecular genetic findings indicated this was a case of ES which unusually appeared to be effectively confined to the bone with no periosteal or extraosseous soft tissue involvement. The patient recovered uneventfully from the operation. Following surgery he underwent adjuvant chemotherapy includes six cycles of VIDT (vincristine, ifosfamide, doxorubicin and toposide). In addition, he had radiotherapy to the left leg, 45 Gy in 25 fractions with boost to the superior and inferior margins to 54.5 Gy (9 Gy in 5 fractions). He made a good functional recovery but developed a proximal non-union of the vascularised fibular graft 18 months after initial surgery, requiring re-excision of the segment of non-union and refixation with reconstruction using an Ilizarov frame. He has been regularly followed-up for 6 years and has shown no evidence of recurrence or development of metastasis.

#### Case 2

**Figure 4:**
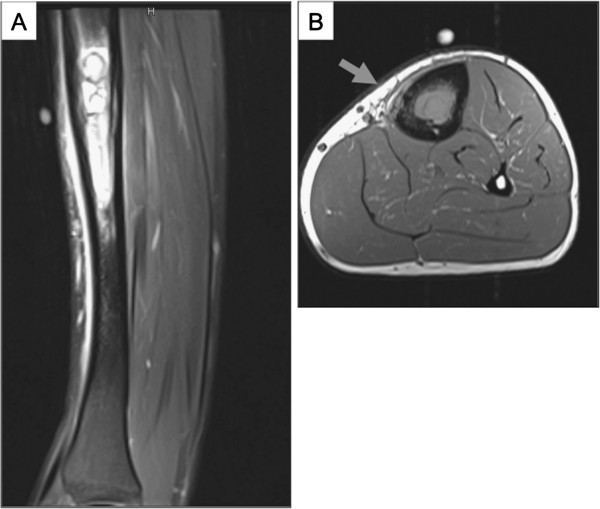
**Case 2: (A) Sagittal image showing a high-signal medullary lesion of the tibial diaphysis. **The linear high signal anterior to the tibia represents reactive oedema. (**B**) T1-weighted axial image showing intramedullary tumour with focal cortical erosion and a minor soft tissue component (arrowed).

The above case of ES was widely discussed with pathology, oncology and surgical colleagues, one of whom (UD) informed us of one other case of ES of the tibia arising in a 16 year old male that had somewhat similar radiological features with extensive bone but limited soft tissue involvement. Radiological features of this case are shown in Figure 4. The biopsy specimen showed a small round cell tumour which had an EWSR1 rearrangement consistent with a (11;22) (q 24;q12). The patient was given neoadjuvant chemotherapy (EURO-E.W.I.N.G. 99). The tumour regressed and histopathology of the resected specimen showed extensive necrosis of the tumour that had extensively involved medullary bone and spread to a limited extent into overlying deep soft tissue. Six years post-treatment the patient is in complete remission.

## Discussion

The two cases of ES documented in this report are unusual in that they show extensive tumour involvement of the tibial bone but limited (Case 2) or no (Case 1) spread of tumour into surrounding soft tissues. Both case 1, which was not treated prior to surgical resection, and case 2, which did receive preoperative chemotherapy, have not shown evidence of recurrence or metastasis after 6 years follow-up; the localized nature of ES in these cases, particularly the limited involvement of extraosseous soft tissue is likely to be significant in regard to this good prognostic outcome.

Most patients with ES present with pain and swelling of the affected bone and surrounding soft tissues. In case 1, there was no clinical evidence of swelling but bone pain had been noted several years prior to diagnosis. No tumour was identified on a plain X-ray when the patient first complained of bone pain. It was only 4 years later when the patient was again investigated that ES was identified. Biochemical and haematology investigations were normal over this 4 year period. Imaging studies showed a large permeative tumour in which there was erosion of cortical bone but no evidence of a significant soft tissue mass or prominent periosteal reaction; the latter is classically onion skinning in type in ES [1,2]. Case 2 also showed extensive medullary involvement and cortical disruption with relatively little soft tissue spread. Histologically, the tumour in case 1 showed typical features of ES. The tumour cells expressed CD99 and molecular genetic investigations confirmed an EWSR1 rearrangement. As in most cases of ES, the tumour largely involved the medulla of the shaft; unusually there was only focal cortical disruption and the tumour was noted to be entirely subperiosteal with no involvement of surrounding soft tissues. No lymphatics are present in bone and this was confirmed in the present case, where there was no evidence of podoplanin expression; lymph node metastases, which can occasionally occur in ES, were not seen in this case but can occur if there is extension of ES outside the bone [5].

There are a few reports of relatively low-grade behaviour of ES, most of which represent rare examples of ES where the tumour is localized to bone and exhibits radiological appearances suggestive of a benign lesion, such as fibrous dysplasia or a simple cyst of bone [6-8]. In one of these reports [6], a series of fibrous dysplasia-like ES of the tibia arising in children was described. Radiologically these cases did not show marked bone destruction and there was little or no periosteal reaction or soft tissue involvement [6]. In case 1 there was permeative lysis of the diaphysis with the proximal and distal margins of the lesion being well defined. Other cases of low-grade ES include a benign cystic lesion of the tibial metaphysis in a 27 year old male [7] and a lytic lesion of the right humerus in a 24 year old who was initially diagnosed as osteomyelitis [9]. In most of the above cases, as in our cases, ES was largely confined to bone and the prognosis was good.

Several clinical and pathological features have been shown to have prognostic value in ES [10-13]. In general, patients younger than 10 years of age have a better treatment response and survival rate than older patients. The tumour site is also thought to be important with distal extremity disease having a better prognosis. Tumour volume has been shown to predict survival with tumours greater than 8 cm in maximum dimension having poorer survival. In both case 1 and case 2, the tibia was affected and tumour volume was relatively high with much of the bone being affected. A significant feature likely to have contributed to the favourable outcome in these cases was that the tumour was effectively confined to the bone compartment. It has been shown that most patients with localized resectable ES (involving both bone and soft tissue) have a relatively good prognosis following surgery and multiagent chemotherapy, with five year survival rates of up to 80% [10,13-15]. Localized disease grossly confined to bone has been reported to have a five year survival rate of 88% compared to 20% with extraosseous extension [15]. Whether risk of progression or relapse associated with non-type 1/non-type 2 EWS-FLI1 fusion is higher than the risk associated with other fusion types is controversial, largely due to the fact that there is a lack of prognostic information on the value of translocation type in a prospectively collected trial cohort [16]. It is unclear in the cases presented whether lack of tumour progression is related to a specific EWS-FLI1 fusion. A number of other biomarkers, such as tumour size, which have been related to poor prognosis in ES, did not appear to correlate with tumour progression in this case [17].

The previously reported cases of ES developing slowly over a period of years, taken with the two cases presented in this report, suggest that there is heterogeneity in the growth pattern of ES with some tumours exhibiting a relatively long intraosseous phase; several of these reported cases were in the tibia [6,7]. Early diagnosis is crucial with regard to disease outcome in ES and other bone sarcomas. The cases presented in this report show that identification of localized ES which has not spread beyond the confines of the bone compartment is important as it is often associated with a good prognosis.

## Competing interests

The authors declare that they have no competing interests.

## Authors’ contributions

All authors read and approved the final manuscript.

## References

[B1] Dorfman H Cerniak B Ewing sarcoma and related entities 1998 St Louis: Mosby 607 642

[B2] de Alava E Lessnick L Sorensen P Fletcher CDM, Hogendoorn PCW, Bridge JA, Mertens F Ewing sarcoma Pathology & Genetics. Tumours of Soft Tissue and Bone 2013 Lyon: IARC Press

[B3] Lopez-Terrada D Molecular genetics of small round cell tumors Semin Diagn Pathol 1996 13 242 249 8875712

[B4] Mirra J Bone tumours: clinical, radiologic and pathological correlation 1989 Philadelphia: Lea and Febiger

[B5] Edwards JR Williams K Kindblom LG Meis-Kindblom JM Hogendoorn PC Hughes D Forsyth RG Jackson D Athanasou NA Lymphatics and bone Hum Pathol 2008 39 49 55 10.1016/j.humpath.2007.04.022 17904616

[B6] Arkader A Myung KS Stanley P Mascarenhas L Ewing sarcoma of the tibia mimicking fibrous dysplasia J Pediatr Orthop B 2011 10.1097/BPB.0b013e32834dfe4d 22094991

[B7] Maheshwari AV Shinault SS Robinson PG Pitcher JD Jr Cystic presentation of Ewing’s sarcoma with indolent clinico-radiologic behaviour Acta Orthop Belg 2009 75 836 841 20166369

[B8] Sundaram M Inwards CY Shives TE Anderson PM Ewing’s sarcoma of the humerus mimicking fibrous dysplasia on imaging and biological behavior Skeletal Radiol 2005 34 285 289 10.1007/s00256-004-0847-x 15838704

[B9] Tow BP Tan MH Delayed diagnosis of Ewing’s sarcoma of the right humerus initially treated as chronic osteomyelitis: a case report J Orthop Surg 2005 13 88 92 10.1177/230949900501300117 15872409

[B10] Bacci G Toni A Avella M Manfrini M Sudanese A Ciaroni D Boriani S Emiliani E Campanacci M Long-term results in 144 localised Ewing’s sarcoma patients treated with combined therapy Cancer 1989 63 1477 1486 10.1002/1097-0142(19890415)63:8<1477::AID-CNCR2820630805>3.0.CO;2-8 2924256

[B11] Kinsella TJ Miser JS Waller B Venzon D Glatstein E Weaver-McClure L Long-term follow-up of Ewing’s sarcoma of bone treated with combined modality therapy Int J Radiat Oncol Biol Phys 1991 20 389 395 10.1016/0360-3016(91)90047-8 1995522

[B12] Mameghan H Fisher RJ O’Gorman-Hughes D Bates EH Huckstep RL Mameghan J Ewing’s sarcoma: long-term follow-up in 49 patients treated from 1967 to 1989 Int J Radiat Oncol Biol Phys 1993 25 431 438 10.1016/0360-3016(93)90064-3 8436521

[B13] Ahrens S Jabar S Heinecke A Prognostic significance of tumour volume in a volume adapated treatment strategy for localised Ewing’s sarcoma of bone Med Pediatr Oncol 1994 23 221 10.1002/(sici)1096-911x(199903)32:3<186::aid-mpo5>3.0.co;2-d 10064186

[B14] Hayes FA Thompson EI Meyer WH Kun L Parham D Rao B Jumar M Hancock M Parvey L Magill L Therapy for localised Ewing’s sarcoma of bone J Clin Oncol 1989 7 208 13 2915236 10.1200/JCO.1989.7.2.208

[B15] Mendenhall CM Marcus JR Enneking WE Springfields DS Thor TL Million RR The prognostic significance of soft tissue extension in Ewing’s sarcoma Cancer 1983 51 913 917 10.1002/1097-0142(19830301)51:5<913::AID-CNCR2820510525>3.0.CO;2-0 6821856

[B16] Le Deley MC Delattre O Schaefer KL Burchill SA Koehler G Hogendoorn PCW Lion T Poremba C Marandet J Ballet S Pierron G Brownhill SC Nesslbock M Ranft A Dirksen U Oberlin O Lewis IJ Craft AW Jurgens H Kovar H Impact of EWS-ETS fusion type on disease progression in Ewing’s sarcoma/peripheral primitive neuroectodermal tumour: prospective results from the cooperative Euro-E.W.I.N.G. 99 trial J Clin Oncol 2010 28 1982 8 10.1200/JCO.2009.23.3585 20308673

[B17] van Maldegem AM Hogendoorn PCW Hassan AB The clinical use of biomarkers as prognostic factors in Ewing sarcoma Clinical Sarcoma Res 2012 2 7 10.1186/2045-3329-2-7 22587879 PMC3351700

